# Impact of Physical Obstacles on the Structural and Effective Connectivity of *in silico* Neuronal Circuits

**DOI:** 10.3389/fncom.2020.00077

**Published:** 2020-08-31

**Authors:** Adriaan-Alexander Ludl, Jordi Soriano

**Affiliations:** ^1^Computational Biology Unit, Department of Informatics, University of Bergen, Bergen, Norway; ^2^Departament de Física de la Matèria Condensada, Universitat de Barcelona, Barcelona, Spain; ^3^Universitat de Barcelona Institute of Complex Systems (UBICS), Barcelona, Spain

**Keywords:** network formation, simulations, patterned networks, structural connectivity, effective connectivity, network bursts, modularity, network measures

## Abstract

Scaffolds and patterned substrates are among the most successful strategies to dictate the connectivity between neurons in culture. Here, we used numerical simulations to investigate the capacity of physical obstacles placed on a flat substrate to shape structural connectivity, and in turn collective dynamics and effective connectivity, in biologically-realistic neuronal networks. We considered μ-sized obstacles placed in mm-sized networks. Three main obstacle shapes were explored, namely crosses, circles and triangles of isosceles profile. They occupied either a small area fraction of the substrate or populated it entirely in a periodic manner. From the point of view of structure, all obstacles promoted short length-scale connections, shifted the in- and out-degree distributions toward lower values, and increased the modularity of the networks. The capacity of obstacles to shape distinct structural traits depended on their density and the ratio between axonal length and substrate diameter. For high densities, different features were triggered depending on obstacle shape, with crosses trapping axons in their vicinity and triangles funneling axons along the reverse direction of their tip. From the point of view of dynamics, obstacles reduced the capacity of networks to spontaneously activate, with triangles in turn strongly dictating the direction of activity propagation. Effective connectivity networks, inferred using transfer entropy, exhibited distinct modular traits, indicating that the presence of obstacles facilitated the formation of local effective microcircuits. Our study illustrates the potential of physical constraints to shape structural blueprints and remodel collective activity, and may guide investigations aimed at mimicking organizational traits of biological neuronal circuits.

## 1. Introduction

Naturally formed biological neuronal networks are characterized by an intricate spatial organization that is central to ensure the functionality of the neuronal circuits (Achard and Bullmore, 2007; Bullmore and Sporns, 2012). The brain's cortex for instance is arranged in columns and hyper-columns that shape structural and functional modules that conduct specialized tasks. The abnormal formation of neuronal circuits during development or their damage due to disease are known to substantially alter circuits' activity patterns. It is therefore well-accepted that the structure of a neuronal circuit shapes its dynamics in great measure. Although a direct relationship between structure and dynamics cannot be established given the intrinsic non-linear nature of neuronal circuits and the coexistence of diverse dynamic physiological mechanisms, there is a wealth of evidence indicating direct correspondences between key structural traits and dynamics (Honey et al., 2010; Sporns, 2011). These traits emerge from general constraints imposed by the spatial embedding of brain circuits (Bullmore and Sporns, 2012; Stiso and Bassett, 2018) together with specific topological characteristics such as high clustering, modularity and the existence of central hub nodes (Sporns, 2011). It has been suggested that these traits and even network motifs can in part be explained from the trade-off between topological integration and the biological cost incurred by nervous systems (Schröter et al., 2017).

In the quest to understand the relationship between structure and dynamics, in particular the importance of structural traits, numerical simulations and *in vitro* studies of neuronal cultures have emerged as invaluable tools. On the one hand, numerical models have been employed to explore various configurations ranging from small-scale circuits (Voges and Perrinet, 2012; Orlandi et al., 2013; Pernice et al., 2013; Faci-Lázaro et al., 2019) to whole-brain dynamics (Honey et al., 2007; Messé et al., 2014; Cabral et al., 2017). Messé et al. for instance used elaborate computational models and anatomical brain data to predict the activity patterns observed in resting-state functional magnetic resonance imaging, and concluded that the backbone of anatomical connectivity strongly shaped overall dynamical traits. Neuronal cultures, on the other hand, have helped elucidate the importance of spatial embedding and imposed metric correlations in shaping spontaneous activity (Orlandi et al., 2013; Hernández-Navarro et al., 2017; Okujeni et al., 2017; Tibau et al., 2020), the impact of modular organization (Shein-Idelson et al., 2011; Tang-Schomer et al., 2014; Yamamoto et al., 2018), the emergence of small-worldness (Downes et al., 2012; de Santos-Sierra et al., 2014), or the role of hubs (Schroeter et al., 2015).

The above studies demonstrated that non-random structural characteristics are central to shape distinct activity patterns and, in turn, specific functional connectivity traits. However, an interesting aspect still to be explored in detail is the impact of definite structural motifs on global network dynamics. This is particularly relevant in the context of *engineered* neuronal cultures (Aebersold et al., 2016), in which the spatial arrangement of neurons and connections is dictated by chemical or physical constraints. Microfabricated structures or *scaffolds* have revolutionized the concept of engineered neuronal cultures by providing both connectivity guidance and structural support to two- and three-dimensional neuronal assemblies (Kunze et al., 2011; Bosi et al., 2015; Severino et al., 2016; Larramendy et al., 2019).

In an effort to help understanding how scaffolds, or specific structural motifs, shape the blueprint, dynamics and effective connectivity of neuronal cultures, we explored numerically small two-dimensional neuronal networks similar to biological *in vitro* ones which incorporated specific scaffold designs in the form of arrays of obstacles. We considered μ-sized scaffolds embedded in a mm-size substrate. Three designs with distinct geometries were explored to examine whether they could imprint specific structural and dynamic features to the networks. The studied obstacles were crosses, circles and isosceles triangles. They were designed to facilitate the trapping or deflection of axons (crosses), to gently modulate connectivity across the network (circles) and to dictate the directionality of connectivity (triangles). We selected these shapes in view of recent experimental studies aimed at guiding neuronal connectivity through microfabrication technology (Crowe et al., 2020). We observed that the obstacles molded structural connectivity at short and long length scales. This induced characteristic features of network dynamics and of effective connectivity. Our study can be extended to tailored designs that mimic specific experimental configurations. Thus, it can improve predictions of the action of scaffolds on living neuronal circuits, for instance to tailor specific dynamic patterns or network functionality.

## 2. Results

### 2.1. Impact of Obstacle Shape on Structural Connectivity

**Figure 1:**
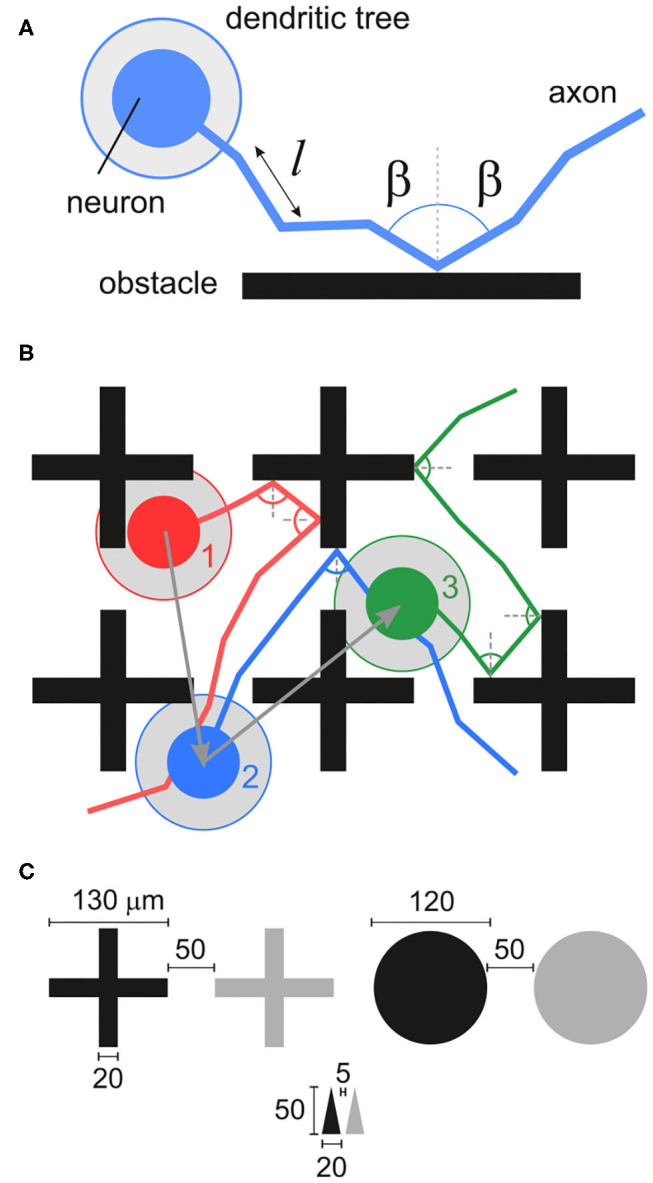
Neuronal network construction. **(A)** Axons are laid out on the substrate by concatenating segments of length *l* that follow a quasistraight path. Whenever the axon hits an obstacle it is deflected with the same angle of incidence. The dendritic tree of the neuron is modeled as a circle (gray area) with a given radius of interaction. **(B)** Neuronal axons in the studied networks interact with obstacles and connect to other neurons. A connection *i*→*j* is established whenever the axon of a neuron *i* crosses the dendritic tree of another neuron *j*. In the sketch, dark gray arrows indicate the connections and their direction, with neurons connecting as 1 → 2 and 2 → 3. **(C)** Sketch of the obstacle geometries used and their dimensions.

**Figure 2:**
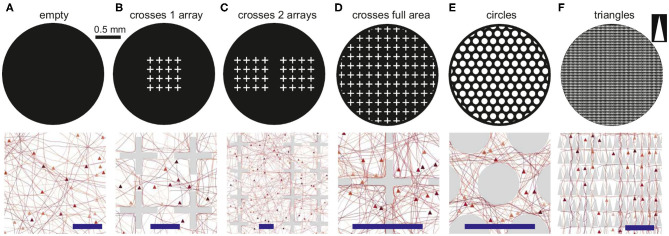
Configurations of obstacles. The top row shows the black and white masks used to set up the simulations, with neurons and connections only placeable in the black areas. The bottom row shows details of the simulated networks, marking the location of neurons (arrowheads) and axons (lines). Obstacles are shown in gray and the blue scale bars are 100 μm. **(A)** Reference empty configuration. **(B,C)** Layouts with 1 array and 2 arrays of crosses. **(D–F)** Layouts of crosses, circles and upwards-pointing isosceles triangles fully covering the substrate.

We explored *in silico* neuronal networks with spatial constraints by considering different sets of obstacles arranged on a circular area of either 2 or 4 mm in diameter. This size was selected to mimic the characteristic size of small *in vitro* cultures (Orlandi et al., 2013; Tibau et al., 2020). In the simulations, neurons were laid out on the surface in a homogeneous manner and connected to one another following a geometric model as in Orlandi et al. (2013), in which the axons grew as concatenated segments according to a biased random walk (Figure 1A) and that is known to mimic well the behavior of individual axons (Feinerman et al., 2008). The presence of obstacles altered axonal growth, an aspect that was modeled by reflecting the axon with the same angle of incidence upon contact with an obstacle (Figures 1A,B). This “reflection rule” was inspired by experimental observations in cultures of physically-constrained neurons (Feinerman et al., 2008; Gladkov et al., 2017) and was the simplest way to introduce interaction with obstacles for this biased random walk. More biologically-accurate models, in which axons may attach to the walls or follow the path of previous axons (Simitzi et al., 2017) were disregarded for the sake of simplicity. We considered three characteristic sets of obstacles, namely crosses, circles and triangles of isosceles profile (Figure 1C), that either occupied a small fraction of the available area or populated it entirely. Table 1 and Figure 2 summarize the different designs chosen and their major characteristics. The density of neurons in the simulations in all configurations was maintained constant at 200 neurons/mm^2^, leading to networks with 625 and 2, 500 neurons for the 2 and 4 mm diameter sizes, respectively.

**Table 1:** Network descriptors for 2 and 4 mm configurations.

	***a*_obs_/*a*_total_(*%*)**	***k*_in_**	***k*_in_**	***k*_out_**	***k*_out_**	**d (mm)**	**d (mm)**
		**μ**	**σ**	**μ**	**σ**	**m**	**s.d**.
**2 mm**							
Triangles	47.2	53.34	22.92	50.21	25.73	0.403	0.306
Circles	44.7	59.08	13.18	54.26	26.26	0.422	0.314
Crosses							
Empty	0.0	66.28	13.83	60.29	33.16	0.533	0.375
1 array	2.4	63.46	15.23	58.21	29.71	0.477	0.346
2 arrays	4.8	60.93	15.06	55.50	28.38	0.446	0.330
Full	13.6	47.98	11.37	45.49	18.07	0.312	0.238
**4 mm (crosses)**							
Empty	0.0	73.51	14.67	67.35	33.97	0.301	0.220
1 array	0.6	72.75	14.37	66.46	34.44	0.293	0.214
2 arrays	1.2	72.20	15.07	65.72	34.63	0.285	0.209
4 arrays	2.4	70.52	15.82	63.37	33.06	0.271	0.202

*For each configuration, we provide the area fraction occupied by the obstacles (a_obs_/a_total_) as well as the average value (μ) and standard deviation (σ) obtained for the Gaussian fits to the distributions of in- and out-degrees (k_in_, k_out_), and the statistical average value (m) and standard deviation (s.d.) of the distribution of connection distances (d)*.

The shape of the obstacles had an important effect on the paths followed by the axons and on the capacity of neurons to connect to one another. The bottom panels of Figure 2 show a detail of the positions of neurons and axons and the interaction of the latter with the obstacles. Crosses (Figure 2D) tended to either deflect axons or to trap them in their vicinity, thus potentially inducing strong local inhomogeneities in the connectivity of the network. Circles (Figure 2E) had a milder effect, deflecting the axons toward the neighborhood, but causing alterations in the connectivity due to the relatively large area that they occupied, reducing the probability of spatially close neurons to interconnect. Finally, triangles shaped as arrowheads pointing upwards (Figure 2F) promoted a strong anisotropy in the connectivity by funneling the axons reverse in the direction opposite to the triangles' tips. This is because axons had a much higher probability to be deflected at the base of a triangle than at its tip. Effectively, as illustrated in Figure 2F, most axons were vertically aligned—although some orthogonal growth remained—and thus neurons tended to connect vertically and downwards.

**Figure 3:**
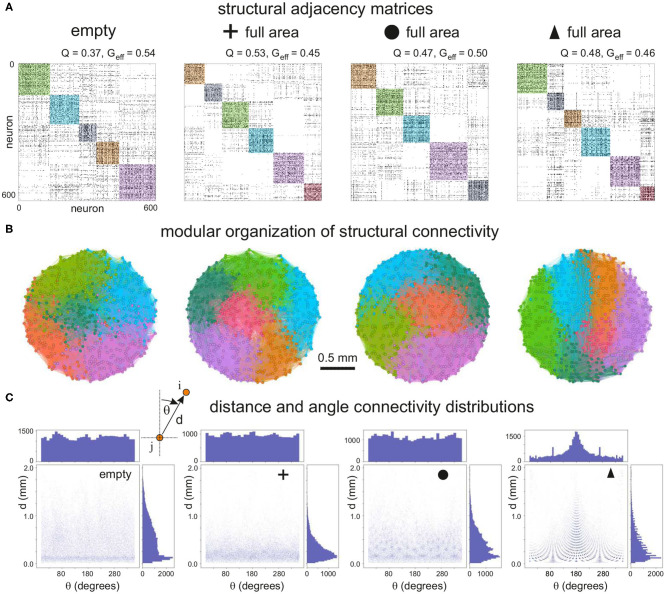
Structural connectivity of 2 mm diameter networks with obstacles. **(A)** Representative connectivity matrices for the “empty” configuration and for networks filled with crosses, circles or triangles. Matrices are arranged to highlight modular structure (colored squares). The values above each matrix indicate the modularity *Q* and the global efficiency *G*_eff_. **(B)** Spatial localization of the identified modules. The color coding matches that of the adjacency matrices. Modularity increases with the presence of obstacles, and the modules are distinctively vertically arranged for triangles. **(C)** Corresponding distribution of connection distances *d* and angles θ between all pairs of connected neurons in each configuration. For triangles there is a characteristic peak at 180°, indicating that most of the neurons connect downwards, i.e., reversed with respect to the triangles' orientation.

To quantify the impact of each configuration on network characteristics we analyzed the topological traits of the resulting structural connectivities. Figure 3A shows representative structural adjacency matrices of the empty configuration together with the configurations made of crosses, circles, and triangles that fully cover the available area. Neuron indices in the matrices are arranged to highlight the existence of communities along the diagonal. We note that communities already appear in the empty configuration (modularity *Q*≃0.37), a trait that is due to the presence of metric correlations in spatially embedded networks (Hernández-Navarro et al., 2017; Faci-Lázaro et al., 2019) which facilitates the formation of local neuronal microcircuits. The global efficiency is relatively high (*G*_eff_≃0.54), indicating that the neurons in the network are well bound together despite spatial effects. The presence of obstacles in the networks in general increased *Q* and decreased *G*_eff_, which reveals a strengthening of metric effects and a reduced capacity for the neurons to connect to one another. The impact of obstacles on structural connectivity depended on their shape. Crosses exhibited the strongest impact, with an increase of *Q* by 43%, while for the other configurations the increase was by 27% (circles) and 30% (triangles). We argue that the trapping of axons caused by the crosses is the cause of the high increase in *Q* for this configuration.

The number and size of structural communities was similar across the panel of configurations. This indicates that neurons were still capable of interconnecting to some degree despite the high spatial density of obstacles. In other words, structural microcircuits emerged but they were not fully isolated. This was verified by analyzing the spatial distribution of the observed communities (Figure 3B), which were physically compact but interlinked. Crosses and circles showed spatial features that were similar to the empty case, with communities appearing in patches of similar shape and size. The triangles configuration, however, shaped communities distinctively organized as vertical stripes and that revealed the strong capacity of triangles to dictate vertical funneling of axons.

To shed light on the impact of obstacles on neuron-to-neuron connectivity and network structure, we investigated the distributions of Euclidean connection distances *d* and angles θ of connections (Figure 3C). For the empty reference case, the distribution of distances was broad, with most of the neurons connecting in the range 0.1−1 mm, although there was a marked peak at *d*≃0.15 mm, a trait again due to the fact that nearby neurons are more likely to connect in spatially embedded networks. For crosses, however, the distribution was strongly shifted toward small connection distances, clearly indicating the capacity of the crosses to trap axons and boost short-range connectivity. Circle and triangle configurations exhibited a behavior in between the previous cases, with broader distributions than crosses but with characteristic peaks that are associated to the size and inter-spacing of the obstacles. On the other hand, the distribution of angles θ was in general homogeneous and similar across configurations except for triangles, with a characteristic peak at θ≃180° associated to the guided top-to-bottom connectivity in the network. Additional peaks appeared at θ≃90° and 270°, which revealed the existence of orthogonal connectivity that facilitated the entire network to be interlinked.

**Figure 4:**
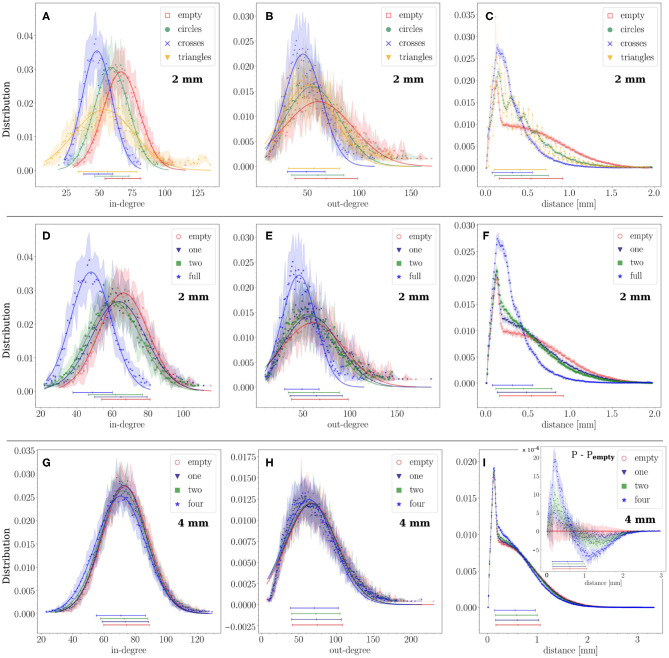
Structural connectivity statistics. **(A–C)** Probability distributions of in- and out-degree (*k*_*in*_, *k*_*out*_) and connection distances *d* for the empty configuration and crosses, circles and triangles configurations that fully cover the substrate. Crosses and triangles exhibit the strongest effect on *k*_*in*_ and *k*_*out*_. **(D–F)** The same distributions for empty, 1 array of crosses, 2 arrays and full coverage in the 2 mm diameter networks. The strongest effect was observed for the configuration in which the crosses fully populated the area, with *k*_*in*_ and *k*_*out*_ distributions shifting to lower values and the distribution of distances exhibiting a marked peak at small length scales. **(G–I)** The same study in a 4 mm diameter network with crosses partially covering the substrate. Empty, 1, 2, and 4 arrays of crosses are compared. The *k*_*in*_ distribution gradually shifts to lower values, and the short distances gain prominence, as the number of arrays increases. The inset of panel **(I)** shows the difference between each distance distribution *P* and that of the empty configuration *P*_empty_. For all distributions and configurations, data is averaged over 12 network replicates. For the distributions of *k*_*in*_ and *k*_*out*_ lines show a Gaussian fit to the data, their parameters are given in Table 1.

To further analyze the impact of obstacles on connectivity, we inspected the distributions of in-degrees (*k*_*in*_) and out-degrees (*k*_*out*_), and also looked in more detail at the distributions of connection distances *d* at different length-scales. The distributions shown in Figure 4 represent averages over 12 replicates for each configuration with the statistical standard deviations shown by the shaded areas. The average value of each distribution and its statistical standard deviation are depicted at the bottom of each graph.

First, we compared the distributions among configurations of circles, triangles and crosses that fully populated the 2 mm substrate (Figures 4A–C). The deviations from the empty configuration were pronounced. Circles, on the one hand, showed *k*_*in*_ and *k*_*out*_ distributions (Figures 4A,B) that lay between those for empty and crosses configurations. This moderate impact contrasted with the existence of periodic peaks in the distribution of distances (Figure 4C). The first peak occurred at 170 μm, which is the distance between the centers of neighboring circles (120 μm circle diameter plus 50 μm separation), and the rest of the peaks are multiples of this typical distance. Thus, circles induced characteristic length scales in the network without strongly altering the degree distributions. Triangles, on the other hand, exhibited a shift of *k*_*in*_ toward lower values and a marked broadening of the distribution. Their effect on *k*_*out*_ was very similar to that of circles. Clearly, the capacity of the triangles to funnel axons along the substrate facilitated long-range connections, whereas the limited orthogonal growth promoted short-range ones. The distribution of distances for triangles (Figure 4C) also shows periodic peaks multiples of 50 μm, the triangle height. These peaks are sharper and steeper for triangles than for circles, the values of which lie in between those for triangles. Crosses showed strong effects as well, which we discuss in detail below.

For crosses, we considered the scenario in which they gradually covered a higher area fraction of the substrate, and compared the empty, 1 array, 2 arrays, and full coverage configurations. As shown in Figures 4D,E, both *k*_*in*_ and *k*_*out*_ distributions gradually shifted toward lower values as the density of occupation increased, although the change was substantial only for full coverage, with the average values of *k*_*in*_ and *k*_*out*_ decreasing by 30%. The distribution of connection distances *d* (Figure 4F) also experienced a strong change for full coverage, with short-range connections dominating the distribution at the expense of highly depleted mid- and long-range ones. These results confirm the hypothesis that crosses either trap axons in a neighborhood or deflect them away, reducing the capacity of neuron to interconnect. The results also reveal that a small occupation of the substrate by obstacles only causes a minor effect in the distribution of connections. This was confirmed by investigating bigger substrates of 4 mm in diameter where the physical dimensions of the crosses were maintained, which thus occupied a very small area fraction (see Table 1). As can be seen in Figures 4G–I, the distributions for the empty, 1, 2 and 4 arrays are very similar to each other and fall within the fluctuations among replicates. Here, the effect of an increase in the number of obstacles is most noticeable on the distance distributions (Figure 4I). As the effect is much smaller than in the previous configurations, we computed the difference between each distribution and that of the empty configuration shown in the inset. It confirms the trend of excess short-range (<0.7 mm) and depleted long-range connections with increased number of obstacles, as seen in the 2 mm configurations. However, these effects are much smaller in the 4 mm case due to the small area fraction occupied by the scaffolds.

An interesting trait of the distribution of distances is the presence of a plateau for the empty case (Figure 4 and Figure S1). This plateau is associated with the broad range of possible axonal lengths, and whose average length (ℓ_*a*_ = 1.1 mm) is an order of magnitude larger than the average radius of the dendritic tree (150 μm), effectively shaping a neighborhood around the neurons in which connection probability is independent of the distance. The presence of obstacles alters this plateau, particularly when they fully cover the substrate, since axons cannot extend freely for long distances.

**Figure 5:**
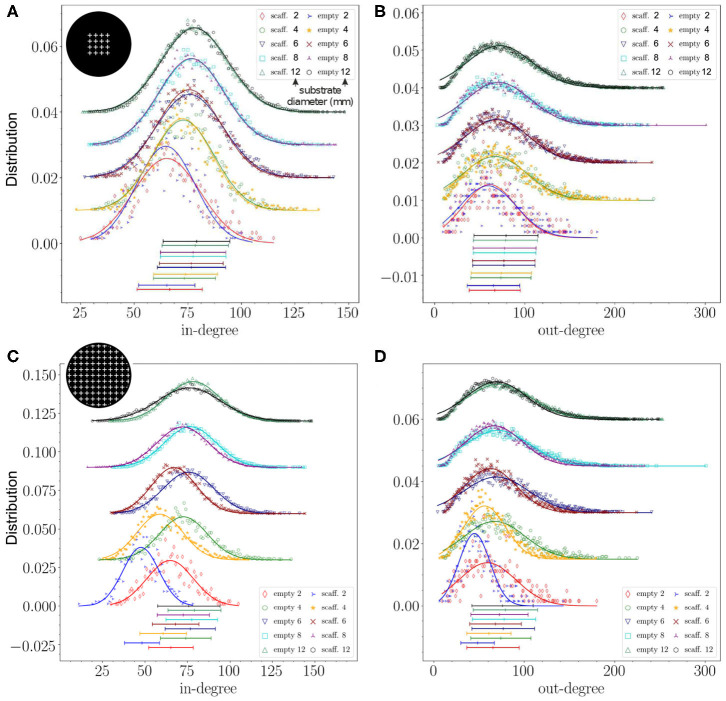
Impact of network size scaling on degree distributions. **(A)** Distributions of in-degree for the empty configuration and for the configuration with 1 array of crosses scaled up to different diameters, preserving the relative size of the scaffolds with respect to substrate diameter. **(B)** Corresponding out-degree distributions. **(C,D)** Distributions of in- and out-degree of the configuration covered by crosses scaled up to different diameters, compared with the empty configuration. For all distributions, effects were more marked for smaller sizes. The data correspond to one representative replicate for each configuration. The lines show a Gaussian fit to the data, their parameters are given in Table 2.

We next explored the effect of substrate size on structural connectivity. We observed that alterations in *k*_*in*_ and *k*_*out*_ degree distributions—relative to the empty configuration—were more prominent when the substrate radius was similar to the characteristic axonal length, approximately 1 mm in our case. This is illustrated in Figure 5, where we compare the degree distributions among 3 networks grown on substrates whose diameters were scaled up from 2 to 12 mm (see Table 2). We considered the empty configuration and the crosses configurations with either 1 array or full coverage. The dimensions of the crosses were also scaled up according to the substrate diameter to preserve the area fraction occupied by obstacles at 2.4% for 1 array and 13.6% for the covered configuration. As shown in panels A and B of the figure, distributions corresponding to the 1-array configuration at different scaling factors were very similar among themselves except for the smallest size of 2 mm diameter, which markedly shifted to low *k*_*in*_ and *k*_*out*_ values. Only for this diameter differences between the empty and obstacles configurations could be appraised. However, for the configuration covered with crosses the effects were stronger as shown in Figures 5C,D. At 2 mm the mean value of *k*_*in*_ decreases by 26.5% and *k*_*out*_ by 24.1% in obstacles compared to empty configurations. At 4 and 6 mm the distributions of both in- and out-degree were still clearly shifted and narrower for the covered configuration. The effect was less clear but still perceptible at 8 and 12 mm scales. We thus conclude that size effects are very important and that they clearly attenuate effects of scaffolds at the area fractions explored here. When the system size is significantly larger than the characteristic axonal length, then metric correlations at short length scales mask out the alterations induced by the obstacles. Nonetheless, obstacles still have an impact on their neighborhood, but from a global perspective the network may appear unaffected.

**Table 2:** Scaled configurations without obstacles (empty), with 1 array of crosses and covered with crosses (full).

	**diameter**	***N*_neur._**	***k*_in_**	***k*_in_**	***k*_out_**	***k*_out_**
	**(mm)**		**μ**	**σ**	**μ**	**σ**
Empty	2	625	64.77	13.67	59.13	31.50
Empty	4	2, 500	72.57	14.02	67.32	33.44
Empty	6	5, 625	75.68	14.92	69.05	36.11
Empty	8	10, 000	76.69	15.18	70.78	35.49
Empty	12	22, 500	78.24	15.57	72.23	36.01
1 array	2	625	65.26	15.60	61.23	29.07
1 array	4	2, 500	72.92	14.49	66.98	35.81
1 array	6	5, 625	75.87	15.69	69.44	34.78
1 array	8	10, 000	77.06	15.25	70.73	35.65
1 array	12	22, 500	77.70	15.48	71.23	35.82
Full	2	625	47.60	10.55	44.89	17.13
Full	4	2, 500	58.78	13.35	56.08	23.17
Full	6	5, 625	67.01	13.25	66.63	31.13
Full	8	10000	72.19	15.14	62.51	28.71
Full	12	22, 500	75.88	18.68	69.26	33.17

*We report the diameter of the configuration, the number of neurons (N_neur._), as well as the average value (μ) and standard deviation (σ) obtained for the Gaussian fits to the distributions of in- and out-degree (k_in_, k_out_)*.

**Figure 6:**
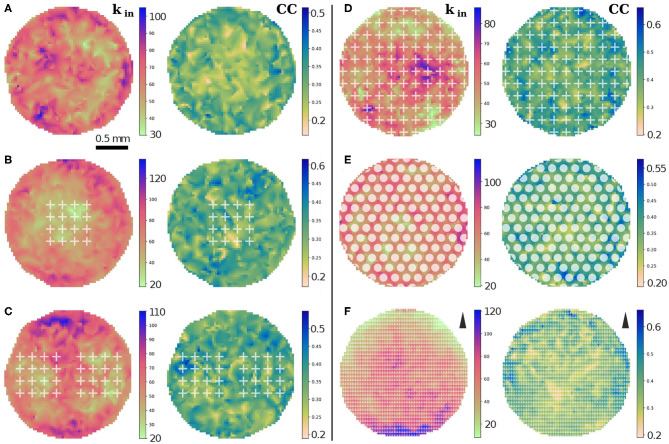
Spatial distributions of in-degree *k*_*in*_ and clustering coefficient CC for the different configurations in the 2 mm substrate. **(A–C)** Distributions for the empty configuration, 1 array and 2 arrays of crosses. The value of *k*_*in*_ decreases by 30% inside the arrays of crosses, while CC is not appreciably affected. **(D–F)** Corresponding distributions for crosses, circles and triangles entirely covering the substrate. Strong effects are observed in the distribution of *k*_*in*_ for crosses and triangles. Compared to the empty configuration, crosses show a decrease of the maximum *k*_*in*_ by 15%; triangles show a gradient of *k*_*in*_ values, which increase in the direction opposite to the tips of the triangles which is indicated next to the colorbar **(F)**. The data correspond to one representative replicate for each configuration.

To complete the analysis of connectivity, we studied the spatial variability in the degree distributions and in clustering coefficients (CCs) in the 2 mm substrate. For sake of simplicity, we considered only *k*_*in*_ in this analysis since it is the distribution that exhibits the strongest differences among configurations. We represented average values of *k*_*in*_ and CC in square regions of side 0.031 mm, containing each about 0.2 neurons. As shown in Figures 6A,C, the empty configuration portrayed strong inhomogeneities in *k*_*in*_ which originated from metric correlations. The addition of obstacles in the form of 1 array or 2 arrays of crosses reduced the in-degree values within the scaffolds and lead to higher values in localized areas outside the scaffolds, hence accentuating inhomogeneities in the network. We note that the *k*_*in*_ distributions shown in Figures 4A,B could not capture these inhomogeneities. Thus, this spatial analysis helps to highlight important fluctuations in the network that cannot be appreciated from solely inspecting the shape of the degree distributions averaged over replicates. The CC values, however, did not show a clear trend in spatial distribution upon the inclusion of scaffolds, although the maximum CCs increased by 20% and tended to concentrated around the scaffolds area, possibly as a consequence of the deflected axons and that facilitated the formation of a higher number of triangles.

The corresponding spatial analysis for obstacles fully covering the network is shown in Figures 6D–F. For crosses, the maximum *k*_*in*_ dropped by 15% relative to the empty configuration and the maximum values appeared concentrated in mostly a few adjacent cells, while patches of low in-degree were more evenly spread across the network. This behavior contrasts with the circles configuration, in which fluctuations among neighboring regions are much weaker, although very high *k*_*in*_ values occur near the border. For triangles, a strong gradient of *k*_*in*_ values emerged that extended across the entire network, with *k*_*in*_ decreasing sixfold in the direction of the tips of the triangles. This patterned distribution of *k*_*in*_ values highlights the strong guidance of the axons, which also favored an increase of the maximum *k*_*in*_ values by 15% compared to the empty configuration. The highest values were localized at the lower edge of the triangles pattern. The CC values for these configurations showed an overall increase of the maximum values by 30% for crosses and triangles, but only increased by 10% for circles. Spatial fluctuations in CCs were marked for crosses and milder for circles and triangles.

### 2.2. Dynamic and Effective Connectivity Alterations Induced by Obstacles

We simulated dynamics of excitatory cortical neurons in the generated structural networks through an integrate and fire model with adaptation, whose parameters were adjusted as in Orlandi et al. (2013) to provide rich spontaneous activity for the empty configuration. Activity was simulated for 30 min for four replicates of each configuration. Then, we explored the changes in collective activity and effective connectivity due to the presence of obstacles in 2 mm diameter cultures which were the ones displaying the strongest effects in the above analyses of structural connectivity. We must note that spontaneous activity comprises both sporadic neuronal activations and network-wide coordinated episodes in the form of network bursts. An abundance of sporadic activations may mask the statistics of network activity and induce artifacts in the analysis of effective connectivity. Thus, in the analysis that follows we filtered out sporadic activity data to emphasize network bursting events, and retained only coordinated activations that encompassed at least 25% of the network.

**Figure 7:**
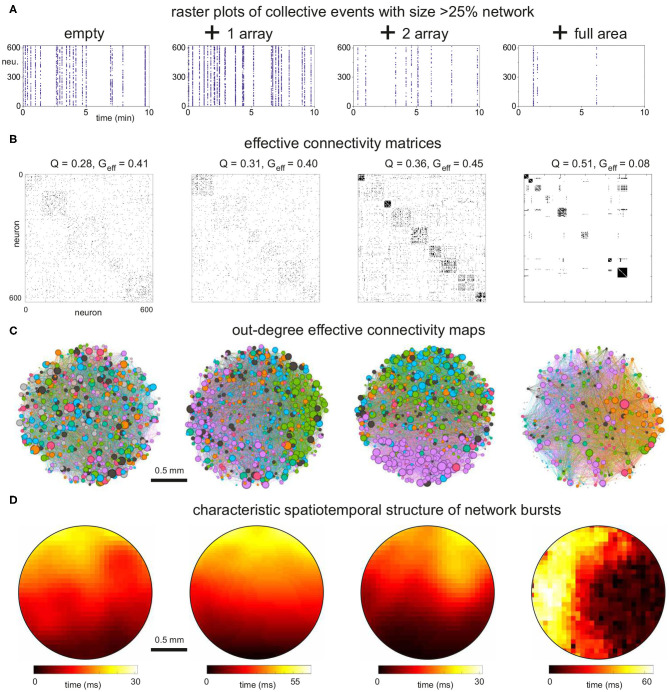
Dynamics and effective connectivity in 2 mm diameter networks with increasing density of cross-shaped obstacles. **(A)** Representative raster plots of bursting events for the empty, 1 and 2 arrays of crosses and full coverage. Bursting activity decreases as the density of obstacles increases. **(B)** Corresponding effective adjacency matrices, with modules along the diagonal. Modularity increases as the density of crosses grows, with a boost by 80% for the full coverage relative to the empty configuration. **(C)** Spatial maps of the effective networks. Neurons are color-coded according to their modularity class, and their diameter is proportional to the out-degree of effective connectivity which reflects activity initiation. Edges are colored according to the outgoing module. The network fully populated with crosses exhibits strong spatial anisotropies. **(D)** Spatiotemporal patterns of representative network bursts. The activity fronts advance as a quasicircular front for the empty and 1 array configurations, to become more structured and erratic for higher densities of crosses. The data correspond to one representative replicate for each configuration.

We first considered the situation in which cross-shaped obstacles progressively populated a larger fraction of the substrate's area. As shown in Figure 7A, network bursting was high for the empty and 1 array configurations, and progressively diminished as the density of obstacles grew. Collective activity almost halted in the configuration in which the obstacles fully populated the area, suggesting that the substantially reduced structural in- and out-degree values strongly affected the capacity of the network to trigger activity and initiate bursts.

The corresponding analysis of the effective connectivity is shown in Figures 7B,C, which provide the adjacency matrices obtained through transfer entropy together with the network maps of community organization and effective out-degree distributions. In the maps, the size of a node is proportional to its out-degree. We chose to plot the out-degree since it reveals the initiation of activity, i.e., which neurons in the network tended to activate other neurons. The adjacency matrix for the empty configuration shows modular traits (*Q*≃0.28) and reveals that some groups of neurons tended to coactivate more frequently with each other than with the rest of the network. The effective modules, however, did not shape compact areas in the network maps but were highly intermixed. This reveals that, despite modularity, network intercommunication was strong as indicated by the high global efficiency (*G*_eff_≃0.41). Activity also initiated in a similar manner throughout the culture, with the highest values of *k*_*out*_ spread out homogeneously.

A similar overall trend was observed for the configuration with 1 array of crosses, which yielded very similar values of *Q* and *G*_eff_. However, the effective modules were more compact and no high out-degree values were observed in the center of the map, where the array is placed, indicating that activity did not initiate within the array. For the 2-array configuration, modularity increased by 25% relative to the empty case, which was accompanied by an increase in the number of modules. This is a sign of higher fragmentation of the dynamics. One of the modules was also compact when represented in the network map (pink-colored neurons), indicating that the obstacles weakened the capacity for whole-network interaction of activity. Most of the activity initiated in this module at the bottom of the map or in small regions at the top, and weak activity was detected within the arrays. These results indicate that the obstacles were capable of shaping effective microcircuits, i.e., a neighborhood of highly activate neurons that poorly interacted with the rest of the network. The isolation of these effective microcircuits strengthen for the configuration in which the crosses fully covered the area (Figures 7B,C, right panels). Here we observed a substantial increase in modularity by about 80% relative to the empty case, with some modules at the verge of full dynamic isolation, as recognized in the effective connectivity matrices by the few links outside the diagonal. *G*_eff_ practically fell to zero, indicating the severely reduced capacity of the network to exchange information. This appears in the map as a large number of disconnected neurons. Activity tended to start at the right edge of the culture (high density of out-degree values), possibly facilitated by the border of the substrate.

To complete the analysis of activity, we also looked at the spatiotemporal structure of network bursts. As shown in Figure 7D, bursting events propagated as circular or quasiflat fronts for the empty and 1 array configurations, reflecting a reduced sensitivity to connectivity inhomogeneities in the network. This neat propagation pattern was altered in the 2-array and full configurations, with propagation showing a richer structure that evinced the strong spatial fluctuations in connectivity.

**Figure 8:**
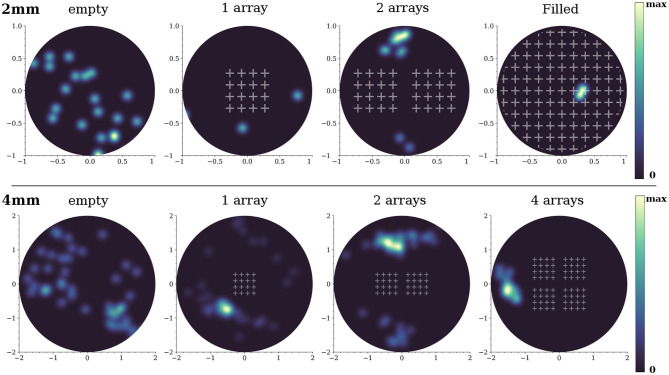
Initiation points for the 2 and 4 mm diameter networks. The blue-yellow patterns are the locations where network bursts commenced. The brighter the color, the higher the occurrence of a burst initiation event in that area. The data shown correspond to one representative replicate of each configuration.

We note that the dynamics in the 2-array and full coverage configurations were very sensitive to the details of the network replicate. We observed that in some instances the simulated networks were incapable of generating network bursts. We characterized this effect on network bursting by computing the spatial distribution of burst initiation events (Figure 8). For the 2 mm diameter network, burst initiation was distributed over most of the area in the empty configuration, but it became increasingly localized as more obstacles were incorporated. Initiation took place outside the scaffolds except for the full coverage configuration, for which the bursting fronts were so fragmented that the identification of initiation could not accurately be determined and most likely occurred near the edges of the network. For comparison, we also provide the results for 4 mm diameter networks. In those simulations the initiation was much richer and the impact of the obstacles was smaller. However, initiation never occurred within the arrays and became more localized as more arrays were added.

**Figure 9:**
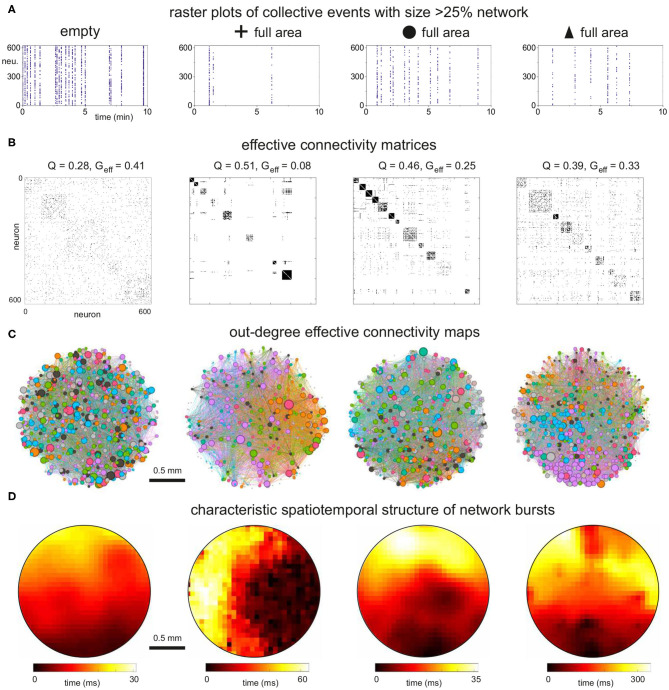
Dynamics and effective connectivity in 2 mm diameter networks filled with obstacles. **(A)** Representative raster plots of bursting events for the empty configuration and full coverage by crosses, circles and triangles. **(B)** Corresponding effective adjacency matrices, with modules highlighted along the diagonal. The modularity *Q* depends on the specific obstacle design, but the number of communities is higher in all cases as compared to the empty configuration. **(C)** Spatial maps of the effective networks. Neurons are color-coded according to their modularity class, and their diameter is proportional to the out-degree of the effective connectivity which reflects activity initiation. Edges are colored according to the outgoing module. While the configuration of circles exhibits traits similar to the empty one, the triangles show a tendency for activity to initiate at the bottom of the network, where *k*_*in*_ is higher. **(D)** Spatiotemporal patterns of representative network bursts. Activity flow is structured for the networks with obstacles. The speed of propagation is a factor 2 and 10 lower for crosses and triangles, respectively, relative to the empty configuration. The data shown correspond to one representative replicate of each configuration.

The equivalent effective connectivity analysis for the different types of obstacles fully covering the substrate is shown in Figure 9. The raster plots compare the characteristic dynamics across configurations. Although all of them displayed decreased activity due to the obstacles, bursting was least affected in circles, mildly in triangles and strongly in crosses, as discussed above. In all cases, however, the effective connectivity matrices (Figure 9B) showed a trend toward high *Q* values relative to the empty configuration which was reflected in an abundance of small sized modules. Circles and triangles, as compared to crosses, exhibited well interlinked modules, with few silent neurons, and therefore their *G*_eff_ values were not as small as in the crosses configuration. The network maps (Figure 9C) illustrate the strong cohesion of the effective networks for circles and triangles, with modules extending all across the area. Effective out-degree values were well spread for circles, indicating that activity initiation equally occurred everywhere. For triangles there was a clear localization of out-degree values toward the bottom of the map, the region that contains also the highest structural *k*_*in*_ values. This correlation between structural and dynamical traits highlights that adequate configurations of obstacles help dictating activity initiation. The structure of spatiotemporal fronts (Figure 9D) shows that all configurations developed structured activity propagation patterns. We point out that the velocity of propagation varied among configurations. Propagating fronts crossed the network in about 30 ms for the empty and circles configurations, while this time increased to 60 ms for crosses and to 300 ms for triangles. The slow propagation observed in triangles is due to the strong connectivity differences between the direction parallel to the triangles' orientation (with high connectivity) and the direction orthogonal to it (weak connectivity), causing the front to advance faster in one direction but slower in the other.

To conclude our study, we compare the major dynamic and network characteristics—structural and effective—among configurations. Figure 9A provides a comparison of the distributions of inter-burst intervals (IBIs), showing the contrasting differences between crosses and the rest of configurations. Figures 9B,C provide the comparison of *Q* and *G*_eff_, respectively. The main plots summarizes the data for the 2 mm diameter networks, while the insets provide the data for the 4 mm ones. All data is organized so that the magnitudes of a given property increase toward the right. For the 2 mm data, the structural network properties varied gently and with very small fluctuations. This contrasts with the effective network properties that exhibited strong changes among configurations and with substantial variability among replicates. For the 4 mm data, all network measures varied gently, either structural or effective, which again highlights the importance of fully covering the substrate with obstacles to induce substantial changes in both structure and dynamics.

## 3. Discussion

**Figure 10:**
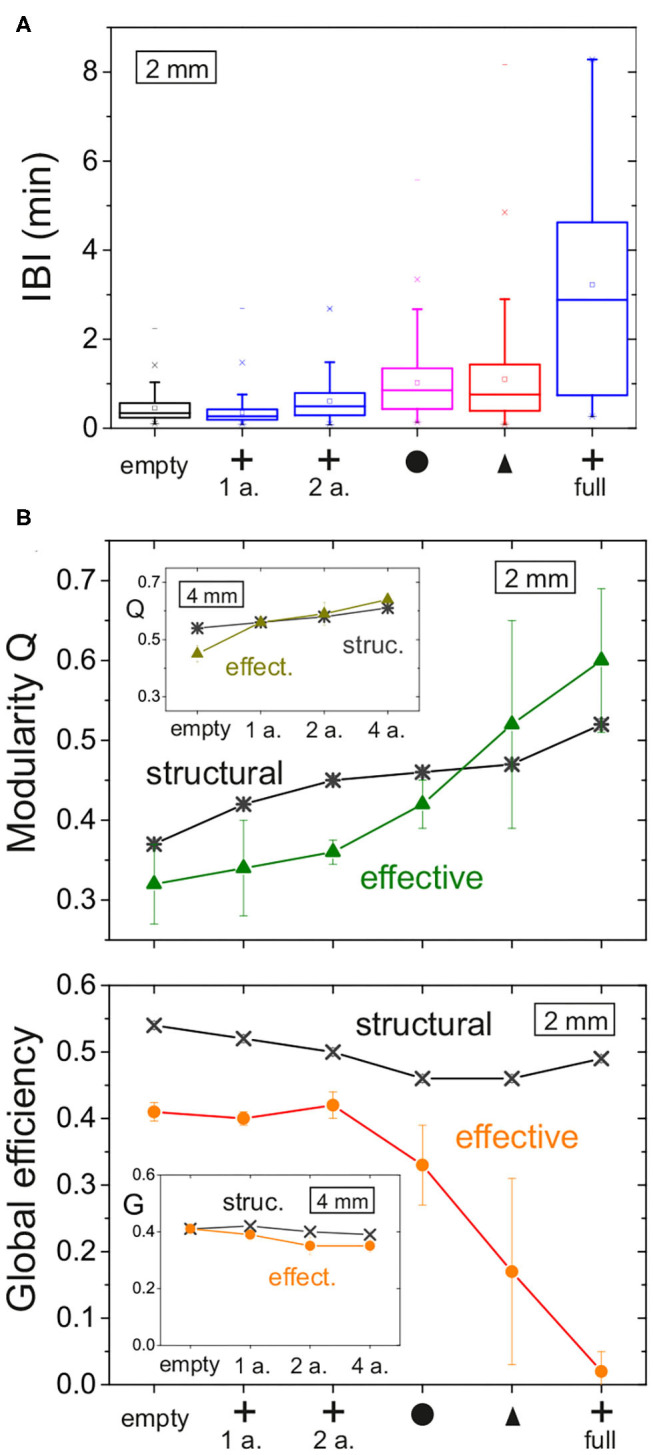
Bursting and effective connectivity statistics for 2 mm networks with obstacles. **(A)** Box plots of the distribution of inter-burst intervals (IBIs) for all the explored configurations. For each box: the inner square is the mean; the central horizontal line the median; the top and bottom box edges are, respectively, the 25th and 75th percentiles; the bottom and top crosses are, respectively, the 1st and 99th percentiles; and the bottom and top dashes are the data range. The IBI in general increases as the density of obstacles grows. Among obstacles fully covering the network, crosses show the strongest alteration on the timing of activity. **(B)** Modularity *Q* and global efficiency *G*_eff_ for the different configurations of obstacles, comparing structural network traits with effective ones. Main plots correspond to networks of 2 mm diameter, and insets to those of 4 mm diameter. Each data point is an average over four replicates, and error bars denote standard deviation.

Our results show that obstacles imprint features on the structural connectivity that may lead to strong alterations in the collective dynamics and effective connectivity of neuronal networks. With those designs of obstacles that we explored, the molding of structural connectivity can occur in two different ways. The first one is by funneling axons in a given direction, as observed with triangles, and the second one is by modifying the spatial density of incoming or outgoing connections in a given region, as observed with crosses. In either case, the capacity of the network to recruit, amplify and propagate activity is affected, thus causing alterations in the timing and spatiotemporal structure of network bursts whose details are sculpted by the underlying structure. When the obstacles fully populated the substrate, their shape was much more important than the total area they occupied. Circles and triangles configurations, both occupying an area fraction of about 45%, caused a twofold increase of the inter-burst interval (Figure 10A), while for crosses the increase was sixfold even though they occupied just 14% of the available area. The capacity of crosses to either trap or deflect axons emerged as a key property as compared to the funneling of axons by triangles or the gentle alteration of axonal paths by circles. In addition to shape, the ratio of typical axonal length to substrate diameter was also a key parameter. When obstacles occupied only a small region of the substrate, as the 1 or 2 arrays of crosses for instance, they induced local alterations whose global effects were masked by the connectivity traits of the rest of the network (Figure 5).

Our simulations reflect the importance of metric correlations in shaping connectivity and dynamics in neuronal circuits. Metric correlations appear naturally in spatially embedded networks (Orlandi et al., 2013; Tibau et al., 2020). As in our simulations, other studies pointed out the spatial distribution of neurons and the characteristic axonal length relative to system size as central ingredients in shaping local and global structural traits (Schmeltzer et al., 2014; Hernández-Navarro et al., 2017; Okujeni et al., 2017). The importance of metric correlations is that they facilitate spatial heterogeneities in the connectivity of the network which greatly influence the dynamic behavior of the entire system, in particular its capacity to initiate and propagate coherent activity in the form of network bursts (Orlandi et al., 2013; Okujeni et al., 2017; Faci-Lázaro et al., 2019). Our work goes a step further and shows that obstacles affect connectivity by changing the shape and average values of in- and out-degree distributions and by altering the range of connection distances, which promoted variations that could be locally very strong. The crosses and triangles configurations were the ones that more significantly altered the spatial structure of connectivity. The in-degree values dropped substantially within areas populated with crosses, while triangles induced a strong gradient of in-degrees along their orientation.

The mechanisms that caused a reduction of the spontaneous activity when obstacles were incorporated are complex. The detailed studies of burst initiation mechanisms by Orlandi et al. (2013) showed that a balance of different network observables was required to maximize bursting, which included in- and out-degrees, clustering coefficients, feed-forward loops and feed-backward loops, among others. Additionally, the study of Orlandi and coworkers pointed out that an excess or deficit of some of these observables could substantially reduce bursting frequency. Our observation that the in- and out-degree distributions are substantially shifted to lower values suggests that they could be major actors in the alteration of activity. This is supported by a recent study of Faci-Lázaro et al. (2019), in which they observed in simulations of neuronal networks similar to ours that the loss of nodes with the highest out-degree precipitated a substantial drop in the number of bursting episodes. The important shift of the out-degree distribution toward lower values for cross-shaped obstacles in Figure 4, much stronger than for other types of obstacles, suggests that out-degree decrease could be one of the most important factors in activity reduction.

We observed that the structural network traits of the studied networks were very similar across network replicates. Even for the configurations in which the obstacles fully populated the substrate, the distributions of *k*_*in*_ and *k*_*out*_ and the values of *Q* and *G*_eff_ varied less than 5% among replicates of the same obstacle design (Figure 10, structural data). However, the effective traits substantially changed as evinced by the large dispersion of both *Q* and *G*_eff_ (Figure 10, effective data). Since the effective connectivity reflects dynamics, the strong contrast between these two network descriptions clearly shows the complex relationship between structure and dynamics, and that the former cannot be directly inferred from the latter with current methods. For instance, the network maps of the configurations with obstacles in Figure 9 are qualitatively similar to one another, but by analyzing only them or the corresponding effective matrices we cannot deduce precisely which structural connectivity or obstacle configuration they emerged from. Thus, our work invites to proceed with caution when trying to infer structural connectivity features from effective ones.

The simulations showed that obstacles increased the modularity in the network, with an impact on both structure and dynamics (effective connectivity). We observed that the impact on structure was similar for all types of obstacles at full coverage of the substrate, with an increase of *Q* by about 30% with respect to the empty case. However, the impact on effective connectivity was much higher, with *Q* increasing by 50% for circles and 80% for crosses. This suggests that the sharp edges of the crosses configuration greatly facilitate the isolation of groups of neurons, a characteristic that is especially relevant for experimental, *in vitro* preparations aimed at enriching the dynamic and functional organization of neuronal networks. For instance, crosses could be placed in groups of four and closer to one another, shaping a structure similar to a hollow square with tiny entrances. Such a structure would create communities of strongly connected neurons with weak connectivity among communities, mimicking for instance the designs of Yamamoto et al. (2018).

Configurations of tailored obstacles could also help shaping networks-of-networks such as the experimentally observed aggregated neuronal networks (Sorkin et al., 2006; Teller et al., 2014) or fractal designs (Díaz Lantada et al., 2013). The latter can be employed to capture the non-Euclidean geometry of the human brain and its relation with developmental traits and multi-scale dynamics (Werner, 2010; Hofman, 2014). Fractality and multi-scale organization are indeed inherent properties of cortical circuits and are closely related to the concept of criticality (Poil et al., 2008; Friedman et al., 2012; Haimovici et al., 2013; Massobrio et al., 2015; Marshall et al., 2016; Johnson et al., 2019), in which a neuronal circuit operates at the boundary between an ordered, strongly coupled state and a disordered, weakly coupled one. Neuronal systems at criticality exhibit long-range spatial and temporal correlations with power-law distributed statistics, facilitating a broad dynamic repertoire and swift communication among distant areas. Massobrio et al. (2015) showed through experiments and theoretical analysis that a critical state can be favored by combining short- and long-range connections, i.e., by imprinting small-world features into a neuronal circuit. Here, we observed that the presence of obstacles, particularly crosses, increased the “small-worldness” (Watts and Strogatz, 1998; Humphries and Gurney, 2008) from 2.46 to 4.03 (SM, Table S1). This confirms that the obstacles trap axons and increase connectivity locally while preserving some long-range connectivity.

Although our simulations aimed at providing a numerical playground to investigate the impact of physical constraints on structural connectivity and dynamics, they were limited by a number of simplifications that could be relaxed in future studies. A first simplification concerns the rule for the growth of axons. We disregarded for simplicity the interaction of axons with neurons or with other axons, and used a simple “reflection” rule to model the interaction between axons and obstacles. *In vitro* experiments in engineered neuronal cultures (Feinerman et al., 2008; Li et al., 2014; Casanova et al., 2018) and microfluidic chambers (Renault et al., 2016; Yamada et al., 2016; Holloway et al., 2019) have shown that axons interact in complex ways with obstacles and that axons often attach to and follow walls. Thus, for a more realistic representation of *in vitro* behavior those interactions should be incorporated in future simulations. A second simplification was the use of excitatory neurons only, which facilitated the inference and analysis of connectivity and its relation with overall network dynamics. The inclusion of inhibition, which typically comprises of about 20% of connections in cortical circuits (Soriano et al., 2008; Schröter et al., 2017), would reduce whole-network bursting and promote a richer spatio-temporal dynamics, as observed experimentally in two-dimensional homogeneous and engineered neuronal cultures (Cohen et al., 2008; Orlandi et al., 2013; Okujeni et al., 2017; Yamamoto et al., 2018). A third simplification was the use of soma and synapse dynamical models that shape cortical-only neuronal networks without plasticity. The inclusion of different cell types and activity-regulatory mechanisms could help investigating questions such as the capacity of the networks to reach activity set points or their response to neuronal loss, as recently explored experimentally *in vitro* (Slomowitz et al., 2015; Teller et al., 2019). And a fourth simplification was the use of solely two-dimensional networks, which only partially reflect the structural complexity and functional richness of naturally-formed brain circuits. Severino et al. (2016) recently showed experimentally and numerically that three-dimensional neuronal networks with fractal organization maintain modular characteristics while promoting long-range connections. As discussed above this facilitates the emergence of a small-world architecture and enhances whole-network bursting. Thus, fractal or three-dimensional patterns could be employed to design more realistic simulations aiming to mimic the dynamic behavior of *in vivo* circuits.

## 4. Conclusion

We have shown that it is possible to dictate the structure of neuronal circuits by incorporating obstacles, whose impact on dynamics and effective connectivity depends on their shape and density. Our work invites the exploration of various configurations in an effort to control the dynamics of the resulting networks. However, achieving precise control remains difficult due to the complex interplay between connectivity, intrinsic neuronal dynamics and noise. Nonetheless, our study provides a method and tools that will allow computational neuroscientists not only to explore a variety of configurations systematically, but eventually contribute to the understanding of the way in which geometry influences the emergence of patterns in growing networks of living neuronal circuits. Thereby, our study can assist in the design of substrates to guide the growth of networks *in vitro*, inviting a quicker and more efficient investigation of prototype geometries than in wet-lab experiments. This will help in finding and selecting suitable candidate geometries for scaffolds or complex architectures in brain-on-a-chip investigations.

## 5. Methods

### 5.1. Geometric Patterns

Three types of obstacles were studied: crosses, circles and triangles. Arrays of obstacles were placed in circular areas of either 2 or 4 mm diameter. Patterns were set as white objects on a black substrate (Figure 2), and simulated neuronal soma and axons were only allowed to grow on the black areas. The neuronal density was set to 200 neurons/mm^2^, leading to networks with 625 and 2500 neurons for the 2 and 4 mm diameter sizes, respectively. An empty configuration with the same number of neurons was also considered as reference (Figure 2A). The different obstacles' geometries are described in detail below.

**Crosses:** The cross-shaped obstacles were 130 μm high and wide, with a beam thickness of 20 μm. The spacing between crosses was 50 μm. They were arranged either in arrays of 4 × 4 crosses, each array covering a square area of side 670 μm, or filling the available substrate entirely (Figures 2B–D). For the latter, a ring 50 μm wide at the edge of the substrate, and free of obstacles, was incorporated to ensure that border effects were the same everywhere in the network. Arrays were placed at the center of the circular substrate. For the 2 mm diameter networks, simulation schemes considered 1 array, 2 arrays, and full occupation; for the 4 mm, simulations considered 1, 2, and 4 arrays. The spacing between arrays was 230 μm. The empty and 1 array configurations were also simulated in a version scaled up by factors 2, 3, 4 and 6. In these scaled versions, the dimensions of the crosses changed according to the scaling factor. The number of neurons placed within the area was scaled to conserve the neuronal density of the smallest configuration (see Table 2).

**Circles:** This design consisted in circles of 120 μm in diameter that were placed in a hexagonal grid covering the entire substrate as shown in Figure 2E. The separation between circles was 50 μm. A 50 μm spacing at the edge of the substrate was incorporated as for the crosses.

**Triangles:** The triangle-shaped obstacles were designed to mimic the geometry of experimental scaffold structures (Crowe et al., 2020). Triangles were of isosceles shape with 50 μm height and 20 μm width. They were placed pointing upwards (Figure 2F). Triangles were arranged in an array that entirely filled the substrate excepted at the edge, that incorporated a ring 5 μm wide free of triangles. The horizontal and vertical separation between triangles at their base was 5 μm.

### 5.2. Network Generation

Neurons were randomly positioned without overlap in the black areas of the designed patterns. Neuronal soma were virtual objects that did not occupy physical space. Thus the axons interacted only with the obstacles and not with the neurons. Neuronal dendritic trees and axons were incorporated following (Orlandi et al., 2013). Briefly, dendritic trees were modeled as circular areas with radius drawn from a normal distribution (mean μ = 150 μm and standard deviation σ = 20 μm), while axons grew at random angles from the neurons' center and followed a biased random walk of concatenated segments of length ℓ (Figure 1), with a total length drawn from a Rayleigh distribution with width σ = 0.9 mm and average axonal length ℓ_*a*_ = 1.1 mm. Upon encountering an obstacle's edge an axon was reflected on the opposite side of the normal to the reflecting surface with a symmetric angle. Once the axons were positioned on the substrate, a connection was established whenever the axon of a given neuron intersected the dendritic tree of any other neuron. The whole network connectivity that resulted from this geometric construction was stored in the *structural* adjacency matrix *S* = {*s*_*ij*_}, where *s*_*ji*_ = 1 corresponds to a connection *i*→*j* and *s*_*ji*_ = 0 otherwise.

### 5.3. Neuron and Synapse Dynamics

A quadratic integrate and fire model with adaptation, based on Izhikevich (Izhikevich, 2003, 2007; Alvarez-Lacalle and Moses, 2009), was used to model the soma dynamics. The equations governing a single neuron are

(1) $$\begin{array}{r} \tau_{c}\frac{d}{dt}v=k\left(v-v_{r}\right)\left(v-v_{t}\right)-u+I+\eta , \end{array}$$

(2) $$\begin{array}{r} \tau_{a}\frac{d}{dt}u=b\left(v-v_{r}\right)-u, \end{array}$$

(3) $$\text{if }v\geq v_{p}\text{ then }v\leftarrow v_{c},\;u\leftarrow u+d_{0}.$$

(4) $$\begin{array}{r} \frac{d}{dt}D=\frac{1}{\tau_{D}}\left(1-D\right)-\left(1-\beta \right)D\delta \left(t-t_{m}\right), \end{array}$$

where the fast soma membrane potential is *v*, the slow inhibitory current is *u*, with τ_*c*_ and τ_*a*_ their respective time constants. The synaptic inputs are denoted by *I*, and the spontaneous emission of spikes is reflected by the noise term η. The resting membrane potential is *v*_*r*_. Above the threshold potential *v*_*t*_, *v* rises to its peak value *v*_*p*_ generating a spike, whereafter it is reset to *v*_*c*_. The membrane potential *u* is reset with the parameter *d*_0_ which describes high threshold conductances. Synaptic depression in Equation (4) is modeled as in Alvarez-Lacalle and Moses (2009), with the characteristic recovery time of synaptic vesicles τ_*D*_ (Cohen and Segal, 2011). Initially, *D* is 1 and after a current injection, i.e., an action potential, at time *t*_*m*_ it decreases as *D* → β*D* with 0 < β <1.

We used the same implementation as in (Orlandi et al., 2013; Tibau et al., 2020). Parameter values were similar to those used in (Orlandi et al., 2013) and were chosen so that the model reproduces typical behavior of cortical neurons. They are also given in the SM (Table S2). Here, all neurons were set to be excitatory for the sake of simplicity. Specifically, we set *g*_AMPA_ and *g*_minis_ equal to 9.5 for all simulations. These values facilitated the generation of network bursts, i.e., activity fronts that encompassed a large fraction of the network, although the timing and spatiotemporal structure of the fronts varied with the obstacles' designs. The time step in all simulations was set to 0.1 ms, with a total duration of 30 min.

### 5.4. Data Processing

#### 5.4.1. Neuronal Activity, Data Filtering and Network Bursts

Simulated networks exhibited rich spontaneous activity that combined sporadic neuronal events with coherent activations of different sizes. Typically, neurons fired either individually or in a coordinated manner at a rate in the range 0.1−0.5 Hz. Since effective connectivity inference was not reliable when sporadic activations were abundant, raster plots of neuronal activity were filtered to retain only coordinated activity episodes. The filtering consisted in computing first the size of coherent network activations in a window of 0.5 s, and next to inspect the distribution of sizes. About 95% of the collective events encompassed at least 25% of the network. Therefore this threshold was chosen to eliminate sporadic activations from the raster plots while only minimally affecting collective bursting episodes. The inter-burst interval (IBI) was then defined as the average time elapsing between two network bursts in which at least 25% of the network participated.

#### 5.4.2. Initiation Points and Representative Spatiotemporal Activity Patterns

Network burst ignition events originated in specific areas of the network, which were termed “initiation points” as introduced in Orlandi et al. (2013). The spatial distribution of these events was obtained by first identifying the starting time of each burst in the raster plots. The neurons in each burst were then reindexed using the time of their first firing during the burst and that provided its spatiotemporal structure in the form of a wave front. This front was fitted to a space-time cone whose apex provided the spatial location of the origin of this burst. Wave fronts that procured coordinates outside the area containing neurons were considered unreliable and excluded. The cone fitting assumed that activity propagated like a circular wave across the network, an assumption that was found valid only for the obstacles' configuration made of crosses. Therefore, the analysis of initiation points was carried out only for this configuration. Given the errors in the cone fitting, the final distributions of initiation points were smoothed versions of the spatially binned histograms of initiation points.

The information about the timing of burst and neuronal reindexing was also used to draw representative spatiotemporal activity patterns. The *x* and *y* coordinates of the neurons participating in the burst were mapped into a grid of 25 × 25 elements. The mapped data was then represented as an smoothed image plot with a color scheme proportional to the propagation time of the burst throughout the network.

#### 5.4.3. Structural and Effective Connectivity

**Structural connectivity:** It corresponded to the ground truth topology that resulted from the geometric construction of the networks. Data was stored in the adjacency matrix *S* = {*s*_*ij*_} which is by construction directed and non-weighted. Their major topological traits were examined using the specified network measures.

**Distributions of connection distances and angles for the structural connectivity:** They were presented as histograms in the figures, and were obtained by combining the information about the spatial location of the neurons and their ground truth topology. The distance *d*_*ij*_ was the Euclidean distance between the centers of the somas of two physically connected neurons *i* and *j*. The corresponding angle θ_*ij*_ was measured as the angle between the vertical axis and the straight line corresponding to the distance *d*_*ij*_.

**Effective connectivity:** It was inferred using a modified version of Transfer Entropy (TE) (Schreiber, 2000). For neurons *X* and *Y* with signals *x*_*n*_ and *y*_*n*_ indexed by 0 ≤ *n* ≤ *n*_max_, where *n*_max_ is the total number of time steps in the data, TE was computed as

(5) $$\begin{array}{rc} {\text{TE}}_{Y\to X}=-\underset{\begin{array}{c} 0\leq n\leq n_{\text{max}}\\ 0\leq k\leq k_{M} \end{array}}{\sum }p\left(x_{n+1},x_{n}^{\left(k\right)},y_{n}^{\left(k\right)}\right) & \times \log_{2}\frac{p\left(x_{n+1}|x_{n}^{\left(k\right)},y_{n}^{\left(k\right)}\right)}{p\left(x_{n+1}|x_{n}^{\left(k\right)}\right)}, \end{array}$$

where *k* is the index of the past time step considered, i.e., the length of the vectors $\left\{x_{n}^{\left(k\right)}\right\}$, and *k*_*M*_ = 2 is the Markov order of the model. Here, instantaneous feedback was assumed, meaning that *X* and *Y* could interact within a time bin, as in Generalized Transfer Entropy (Stetter et al., 2012; Orlandi et al., 2014). Thus, the Markov order superscript indices on $\left\{x_{n}^{\left(k\right)}\right\}$ and $\left\{y_{n}^{\left(k\right)}\right\}$ are identical. This assumption was justified because the synaptic time constants (≃ 1 ms) were much smaller than the time bins (50 ms) used. This binning also ensured that data analysis was feasible and reasonably fast. Effective connectivity was inferred for 30 min long raster plots (*n*_max_ = 36, 000) containing network bursting events only. For any connection *X* to *Y*, significance *z* was established by comparing the TE_*Y*→*X*_ estimate with the joint distribution of TE for all input scores *X*′ to *Y* and output scores *X* to *Y*′ (for any *X*′ and *Y*′), as

(6) $$\begin{array}{r} z=\frac{{\text{TE}}_{Y\to X}-\langle {\text{TE}}_{\text{joint}}\rangle }{\sigma_{\text{joint}}}, \end{array}$$

where 〈TE_joint_〉 is the average value of the joint distribution and σ_joint_ is its standard deviation. Significant connections were then set as those with *z* ≥ 2. This threshold was considered optimal since it captured the flow of neuronal communication during activity at both local and global scales. A lower threshold of *z* = 1 yielded networks that excessively emphasized whole-network coordinated activity, effectively shaping random graphs in all studied cases. Thresholds *z*≳3 emphasized the strongest neuron-to-neuron interactions and often yielded empty matrices. Significant connections were finally thresholded to 0 (absence of connection) and 1 (presence of connection). The final effective connectivity matrices **E** were then directed and non-weighted.

#### 5.4.4. Network Analysis and Measures

The following network statistics and centrality measures were computed for both structural (**S**, ground truth) and effective (**E**) topologies.

**In- and out-degree distributions and clustering coefficient:** Degree statistics were computed in Python using the Brain Connectivity Toolbox (BCT) (Rubinov and Sporns, 2010). For the structural connectivity, these distributions reflected the capacity of the obstacles to shape or dictate a distinct circuitry. For the effective connectivity, they reflected the flow of activity. Clustering coefficients (CC) (Fagiolo, 2007) were computed using the Python module NetworkX (Hagberg et al., 2008). The spatial distributions represented as heatmaps in Figure 6 show the average values of in-degree (*k*_*in*_) and CC in square regions of side 0.031 mm, containing ≈ 0.2 neurons on average for the networks of 2 mm in diameter. Therefore, linear interpolation was used to improve readability of the heatmap. For larger network sizes, the size of the squares was scaled up proportionally to the diameter of the network.

**Modularity** **Q:** It quantified the likelihood that neurons were organized in communities, i.e., that neurons within a community were more connected with themselves than with neurons in other communities. Following Rubinov and Sporns (2010), *Q* was computed as

(7) $$\begin{array}{r} Q=\frac{1}{2m}\underset{0\leq i,j\leq N}{\sum }\left(A_{ij}-\frac{k_{i}k_{j}}{2m}\right)\delta \left(c_{i},c_{j}\right), \end{array}$$

where *N* is the number of neurons, *A*_*ij*_ represents the weight of the connection between *i* and *j*, $k_{i}=\overset{N}{\underset{j=1}{\sum }}A_{ij}$ is the sum of the weights of the connections attached to neuron *i*, *c*_*i*_ is the community to which neuron *i* belongs, $m=\frac{1}{2}\overset{N}{\underset{i,j=1}{\sum }}A_{ij}$, and the δ(*u, v*) function is 1 for *u* = *v* and 0 otherwise. Optimal community structure was computed using the Louvain algorithm (Blondel et al., 2008). *Q* ranged from 0 to 1, with *Q* ≈ 0 for a random, non-modular network and *Q* → 1 for a strong modular organization.

**Global efficiency** ***G***_**eff**_**:** It quantified the integration capacity of the network, i.e., the performance of information exchange among neurons across the network. It was calculated using the BCT. Following (Latora and Marchiori, 2001; Rubinov and Sporns, 2010), the efficiency *E* of a network of *N* nodes was computed as

(8) $$\begin{array}{r} E=\frac{1}{N\left(N-1\right)}\underset{0\leq i,j\leq N}{\sum }\frac{1}{\lambda \left(i,j\right)}, \end{array}$$

where *N* is the number of neurons and λ(*i, j*) is the length of the shortest path connecting neurons *i* and *j*. The global efficiency *G*_eff_ is the relative value *G*_eff_ = *E*/*E*_id_, where *E*_id_ refers to the efficiency of an ideal graph that has all *N*(*N*−1) possible connections.

## Data Availability Statement

The raw data supporting the conclusions of this article will be made available upon request by the authors, without undue reservation. Source code pertaining to certain aspects of the analysis is available at https://github.com/adluinf/SpatialNetworkAnalysis.

## Author Contributions

A-AL designed and performed the simulations. All authors analyzed the data, contributed to the interpretation of results, and writing of the article.

## Conflict of Interest

The authors declare that the research was conducted in the absence of any commercial or financial relationships that could be construed as a potential conflict of interest.
