# Supplementary material for: Impact of Physical Obstacles on the Structural and Effective Connectivity of in silico Neuronal Circuits
Source: Front Comput Neurosci. 2020 Aug 31;14:77. doi: 10.3389/fncom.2020.00077 (PMC7488194; doi:10.3389/fncom.2020.00077)
Supplement: Supplementary file 1 [file Data_Sheet_1.pdf]

Supplementary Material for

Impact of Physical Obstacles on the Structural and Effective Connectivity of in silico *Neuronal Circuits*

A.-A. Ludl & J. Soriano, Front. Comput. Neurosci. 14:77 (2020). DOI: 10.3389/fncom.2020.00077.

Here we provide additional analyses illustrating the effect of different network generation parameters on structural connectivity in the 'empty' configuration, i.e. without obstacles. We also provide two additional tables, a first one for the values of the small–worldness analysis of the structural connectivity, and as second one containing all the simulation parameters used.

1 EFFECTS OF PARAMETERS ON NETWORK GENERATION

We briefly present the impact of parameters controlling the random walk of the axon, the size of the dendritic tree and the neuron density on the network measures (in– and out– degree, connection distance).

Figure S1 shows the probability distributions of in– and out–degree (k_{in}, k_{out}) and connection distances d for configurations generated in an empty circle of 2 mm diameter following the organisation of Figure 4 of the main text. For each configuration the data show the mean and standard deviation of 12 realisations computed with the same set of parameters.

For panels **(A)**-**(C)** the parameter controlling the axonal length was varied from 0.45 to 1.8 mm. It can be seen that the in– and out–degree distributions become broader and shift to higher values as the axonal length becomes larger. The distance distributions also broaden, with the shoulder present for short axonal lengths extending first to the plateau seen at intermediary lengths as in Figure 4 of the main text, then becoming a second peak at about 0.9 mm for the largest axonal lengths shown. It is important to note that the axonal length is a random variable for which a value is drawn for each neuron from the Rayleigh distribution.

For panels **(D)**-**(F)** the parameter controlling the spread of the random angle at each step of the axon's random walk was varied from 0.4 to 6.2 radians. In the range explored this parameter has little effect on the distributions shown. Only for the smallest values do we see a small shift in the in– and out–degree distributions. For the smallest values we also see that the plateau in the distance distribution observed for the standard parameters is replaced with a nearly linear drop between approximately 0.2 and 1 mm.

For panels **(G)**-**(I)** the parameter controlling the radius of the dendritic tree was varied from 75 to 300 μm. It can be seen that the in– and out–degree distributions become broader and shift to higher values as the dendritic tree becomes larger. This parameter affects the first peak of the distance distribution, which broadens and shifts to larger values as the radius of the dendritic tree grows.

For panels **(J)**-**(L)** the density of neurons was varied from 100 to 400 neurons/mm^2. It can be seen that the in– and out–degree distributions become broader and shift to higher values as the neuron density becomes larger. This parameter does not visibly affect the distance distribution.

Table S3 gives values of the fit parameters for in– and out–degree distributions, as well as the mean and standard deviation for the distance distribution for 2 mm configurations with varying parameters.

Table S1. Small-worldness for 2 mm diameter networks with different obstacle designs fully populating the substrate. Data are averaged over 4 network realizations and are shown as mean and standard deviation (sd) of the mean.

	S^{SW} mean	S^{SW} sd
empty	2.46	0.07
circles	3.13	0.02
triangles	3.16	0.06
crosses	4.03	0.06

1.1 Small–worldness

Following Watts and Strogatz (1998), a network G with N nodes and M edges is a small–world network if it has a similar path length L but greater clustering coefficient C of nodes than an Erdös-Rényi (ER) random graph with the same number of nodes and links. To assess whether these criteria are satisfied, we employ the small–worldness S^{WS} as introduced in Humphries and Gurney (2008):

$$S^{\mathrm{WS}} = \frac{\gamma^{\mathrm{WS}}}{\lambda}, \tag{S1}$$

where $\gamma^{\mathrm{WS}} = C^{\mathrm{WS}}/C^{\mathrm{rand}}$ is the ratio between the clustering coefficients of network G as defined by Watts and Strogatz (1998) C^{WS} and its random graph equivalent C^{rand}, and $\lambda = L/L^{\mathrm{rand}}$ is the ratio between the shortest path length L of network G and its random graph equivalent L^{rand}. Thus, small–worldness is satisfied if $\gamma^{\mathrm{WS}} \gg 1$ and $\lambda \geq 1$, i.e. $S^{\mathrm{WS}} > 1$. The larger S^{WS} deviates from 1, the stronger the small–world effect is. We note that other definitions of clustering, and therefore small–worldness, can be used (Humphries and Gurney, 2008).

For our study, we considered the S^{WS} definition and computed it on the generated structural connectivity matrices using the MATLAB libraries provided by Humphries and Gurney (2008). Table S1 summarizes the results obtained for the 2 mm diameter networks with different configurations of obstacles that fully populate the substrate.

1.2 Simulation Parameters

Table S2 gives the values of the model parameters for the simulations presented in the main text of this article. The model is based on that presented in Orlandi et al. (2013).

REFERENCES

Watts DJ, Strogatz SH. Collective dynamics of ‘small-world’ networks. *Nature* **393** (1998) 440–442. doi:10.1038/30918.

Humphries MD, Gurney K. Network ’small-world-ness’: A quantitative method for determining canonical network equivalence. *PLoS ONE* **3** (2008). doi:10.1371/journal.pone.0002051.

Orlandi JG, Soriano J, Alvarez-Lacalle E, Teller S, Casademunt J. Noise focusing and the emergence of coherent activity in neuronal cultures. *Nature Phys* **9** (2013) 582–590. doi:10.1038/nphys2686.

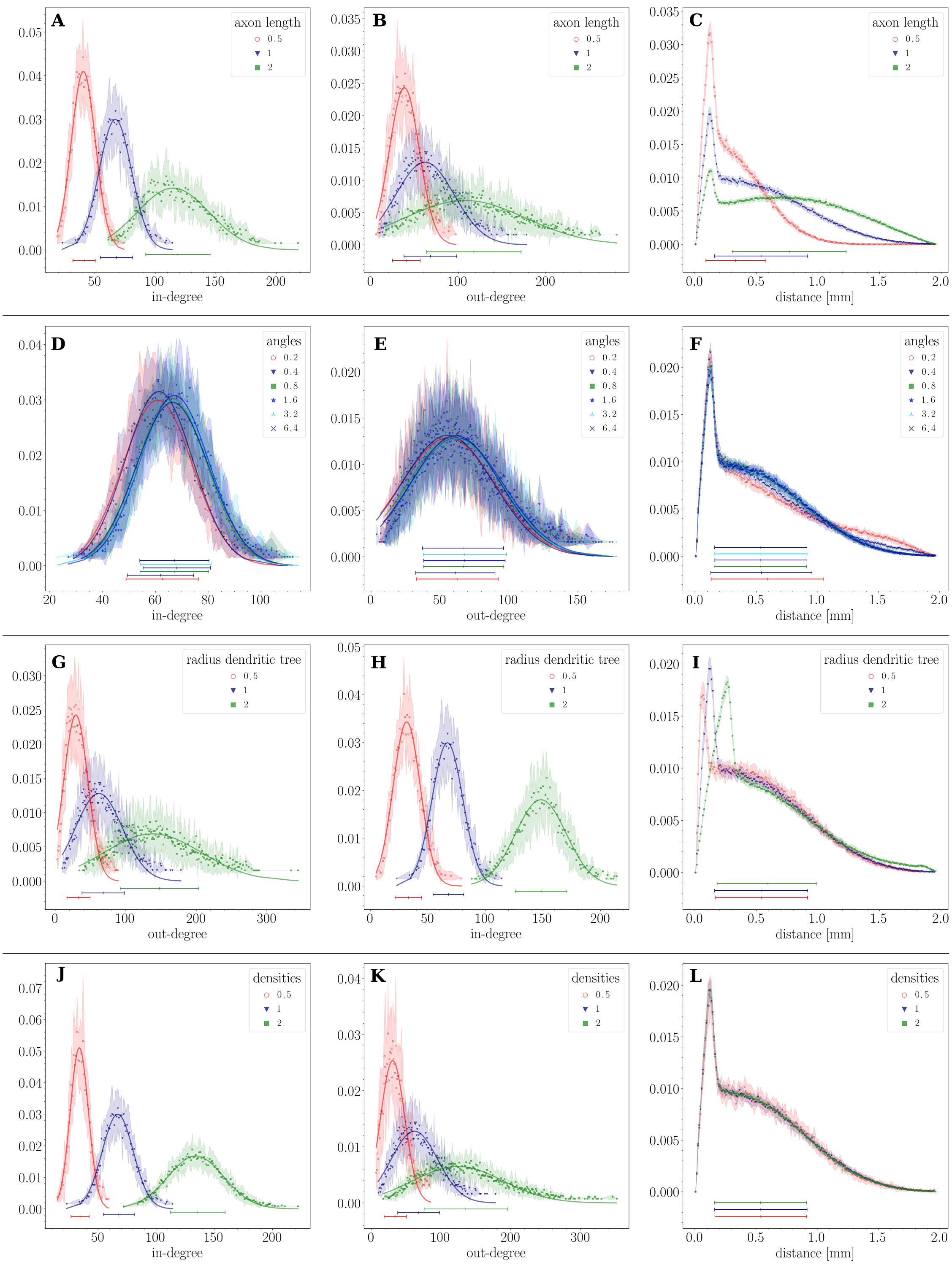

Figure S1. Effect of parameters on structural connectivity statistics. Each row presents the probability distributions of in– and out–degree (k_{in}, k_{out}) and connection distances d for configurations generated in an empty circle of 2 mm diameter. **(A)-(C)** Distributions obtained when varying the axon length. **(D)-(F)** Distributions obtained when varying the range of angles for the axon's random walk. **(G)-(I)** Distributions obtained when varying the radius of the dendritic tree. **(J)-(L)** Distributions obtained when varying the density of neurons. This figure follows the organisation of (Figure 4). For all distributions and configurations, data is averaged over 12 network replicates. For the distributions of k_{in} and k_{out} lines show a Gaussian fit to the data, their parameters are given in **Table S3**.

Table S2. Parameters and values for the model.

Parameters	Symbol	Value	Unit
System-wide parameters			
System radius	r	$2-4$	mm
Density	ρ	200	neur./mm^2
Morphological parameters			
Soma size (fixed)	r_a	7.5	μm
Dendritic tree (Gaussian pdf)	(μ, σ)	$(150, 20)$	μm
Axonal length (Rayleigh pdf)	σ	900	μm
Axonal segment length (fixed)	l	10	μm
Axonal segment angle (G. pdf)	$(\mu, \sigma, \sigma_{\max})$	$(0, 0.1, 3.2)$	rad
Soma parameters			
Resting membrane potential	v_r	-60	mV
Threshold membrane potential	v_t	-45	mV
Peak membrane potential	v_p	35	mV
Reset membrane potential	v_c	-50	mV
	τ_c	50	ms
	k	0.5	mV^{-1}
	τ_a	50	ms
	b	0.5	
	d_0	50	mV
Synapse parameters			
Depression recovery time	τ_D	3000	ms
Depression decay	β	0.8	
AMPA current strength	g_A	9.5	mV
AMPA current decay time	τ_A	10	ms
Noise parameters			
White noise strength	g_s	300	mV^{2}ms
White noise auto-correlation	$\langle \eta(t)\eta(t') \rangle$	$= 2g_s\delta(t-t')$	
Shot noise frequency	λ	0.166	ms^{-1}
Shot noise strength (minis)	g_m	$= g_A$	mV
Shot noise decay time	τ_m	$= \tau_A$	
Simulation parameters			
Algorithm			2nd order Runge-Kutta
Time step	Δt	0.1	ms
Typical Run time		30	min

Table S3. Network descriptors for 2 mm configurations with varying parameters. The following parameters were varied: the parameter $\sigma_{\mathrm{ax.len.}}$ for the axonal length, the maximal cumulative angle allowed for the axonal random walk ($\alpha_{\max}$) the radius of the dendritic tree ($r_{\mathrm{d.t.}}$), and the density of neurons (ρ). For each case, we provide the average value (μ) and standard deviation (σ) obtained for the Gaussian fits to the distributions of in– and out–degrees (k_{in}, k_{out}), and the statistical average value (m) and standard deviation (s.d.) of the distribution of connection lengths (l). These parameters correspond to the data shown in Figure S1.

	in-degree		out-degree		length	
	k_{in} μ	k_{in} σ	k_{out} μ	k_{out} σ	l (mm) m	l (mm) s.d.
$\sigma_{\mathrm{ax.len.}}$ (mm)						
0.45	40.45	10.02	38.25	17.09	0.330	0.241
0.9	66.95	13.43	61.89	33.45	0.538	0.378
1.8	114.70	29.96	107.68	69.19	0.766	0.462
$\alpha_{\max}$ (rad.)						
0.2	60.88	13.24	55.75	34.45	0.591	0.461
0.4	61.39	12.81	55.57	33.36	0.542	0.413
0.8	66.26	13.33	60.47	34.41	0.533	0.378
1.6	67.21	13.19	62.20	34.00	0.537	0.377
3.2	66.95	13.43	61.89	33.45	0.538	0.378
6.4	66.49	13.76	61.34	32.70	0.537	0.378
$r_{\mathrm{d.t.}}$ (μm)						
75	31.54	11.83	29.17	17.15	0.541	0.373
150	66.95	13.43	61.89	33.45	0.538	0.378
300	148.18	22.07	138.10	68.90	0.586	0.405
ρ (neur./mm^2)						
100	33.85	7.83	30.72	17.25	0.538	0.374
200	66.95	13.43	61.89	33.45	0.538	0.378
400	134.14	24.12	124.34	64.79	0.536	0.376
