# Supplementary figures and images for: Impact of Physical Obstacles on the Structural and Effective Connectivity of in silico Neuronal Circuits
Source: Front Comput Neurosci. 2020 Aug 31;14:77. doi: 10.3389/fncom.2020.00077 (PMC7488194; doi:10.3389/fncom.2020.00077)

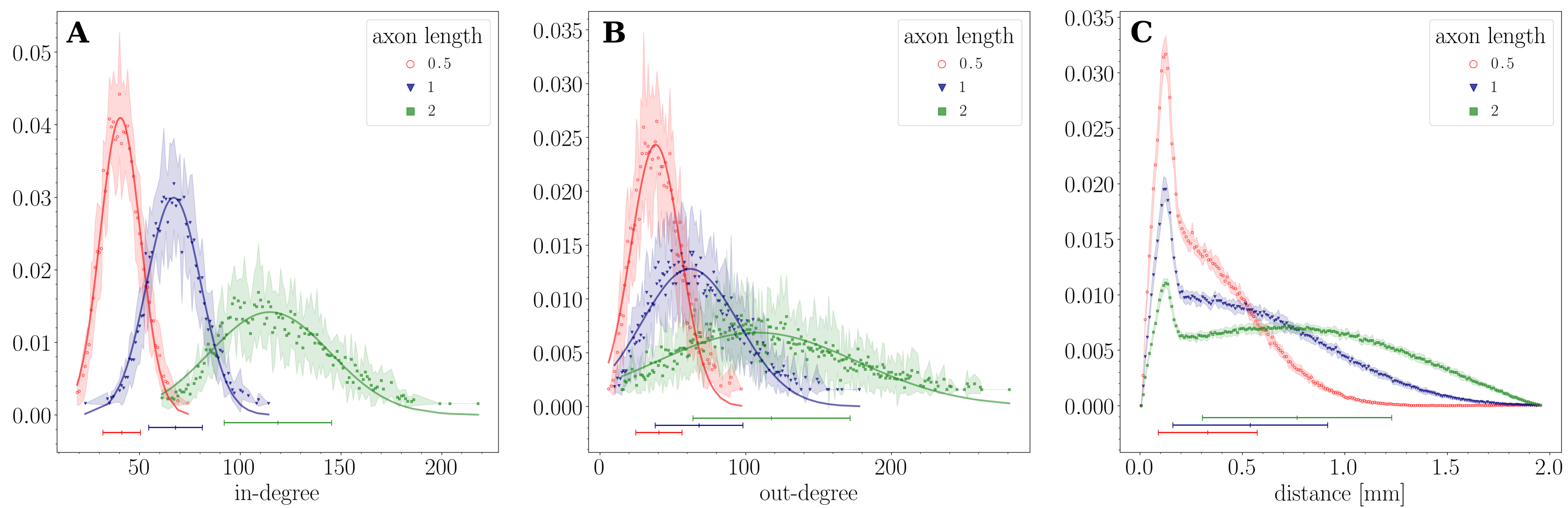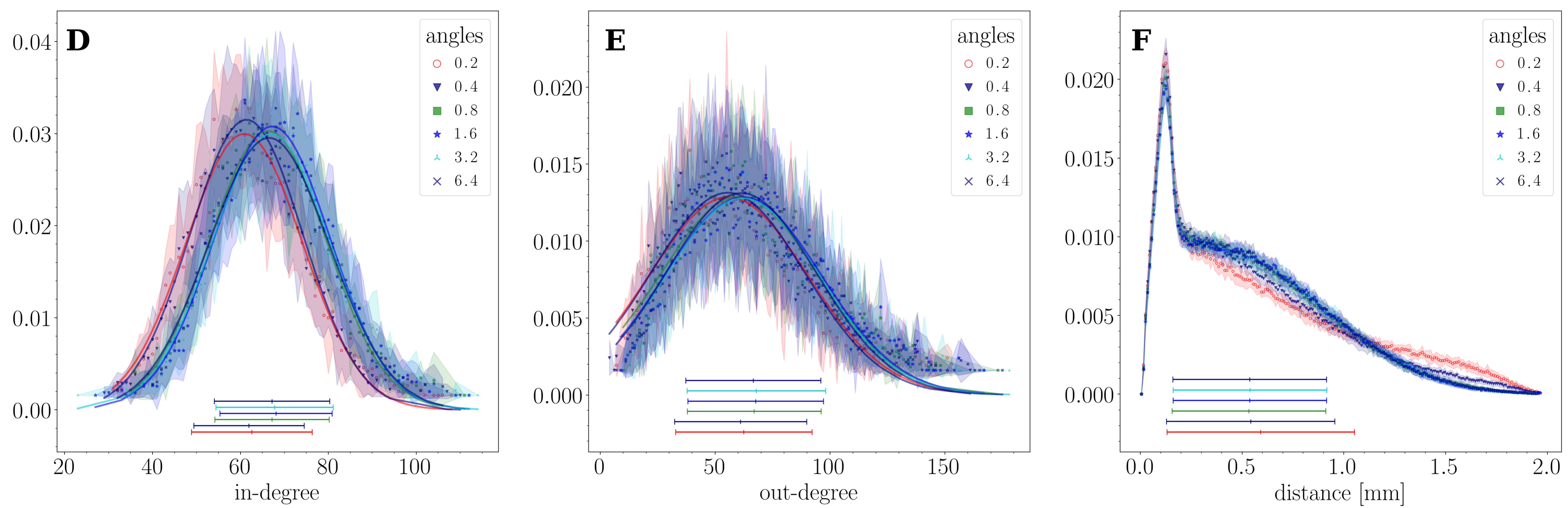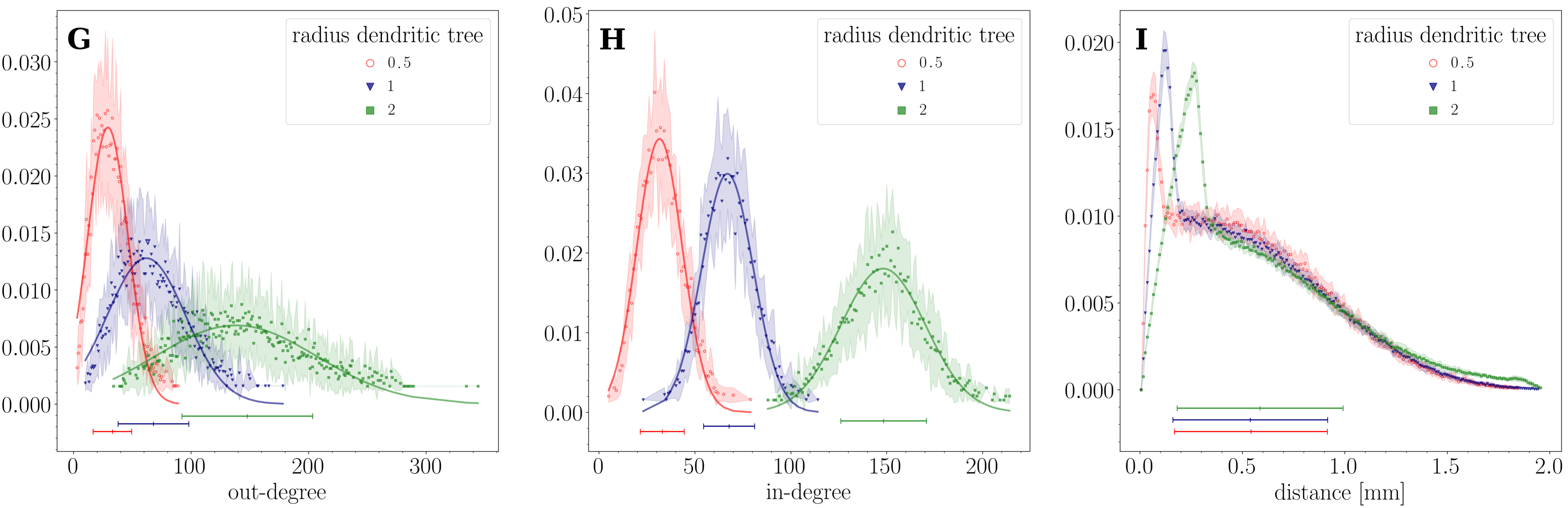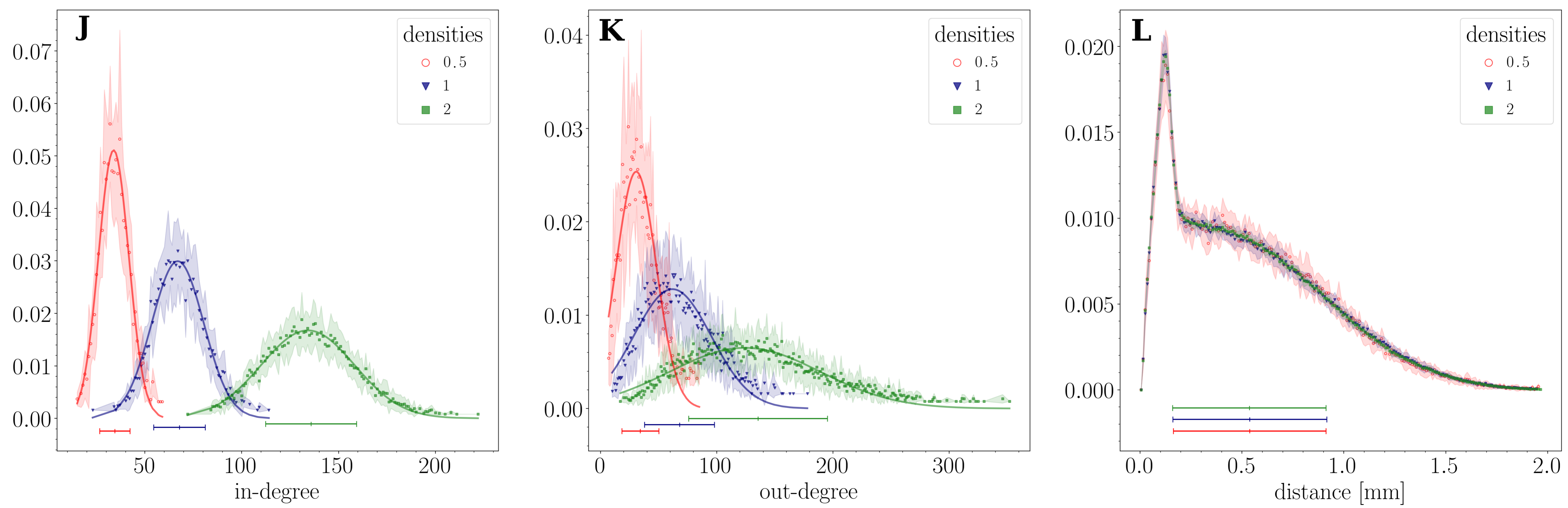

Supplement: Supplementary file 2 [file Data_Sheet_1.zip › Supplement_Figure_1.pdf]
